# A novel intermediate host for *Taenia serialis* (Gervais, 1847): The European roe deer (*Capreolus capreolus* L. 1758) from the Monti Sibillini National Park (MSNP), Italy

**DOI:** 10.1016/j.ijppaw.2021.12.011

**Published:** 2021-12-29

**Authors:** Benedetto Morandi, Alessandra Bazzucchi, Sofia Gambini, Silvia Crotti, Deborah Cruciani, Federico Morandi, Maira Napoleoni, Toni Piseddu, Alessandra Di Donato, Stefano Gavaudan

**Affiliations:** a Istituto Zooprofilattico Sperimentale Dell’Umbria e Delle Marche “Togo Rosati”, Via G.Salvemini, 1, 06126, Perugia, Italy; b Monti Sibillini National Park, P.zza Del Forno 1, 62039, Visso, Italy; c OIE Reference Laboratory for Echinococcosis, National Reference Laboratory for Cystic Echinococcosis (CeNRE), Istituto Zooprofilattico Della Sardegna (IZS), Via Vienna 2, Sassari, 07100, Italy

**Keywords:** Wildlife, Parasites, Metacestodes, Taenia serialis, *Capreolus capreolus*, Monti sibillini national park

## Abstract

Taeniids are multi-host parasites with an indirect life cycle that strictly depends on a predator-prey relationship. Parasites with a complex life cycle may exhibit different degrees of host-specificity at each life stage. Knowing the host breadth is a fundamental concept of the biology and epidemiology of these multi-host parasites. Morphological identification of tapeworms is challenging and occasionally may produce misdiagnosis. Thus, molecular investigations were carried out for the identification of parasitic cysts detected from muscle tissues in a male roe deer necropsied at the Istituto Zooprofilattico Sperimentale dell’Umbria e delle Marche “Togo Rosati” (Central Italy). Sanger sequencing showed 99% query cover, 2e-109 e-value, and 100% identity with *Taenia serialis*. The exact definitive host was not revealed in this report, but red foxes and Italian wolves may play a significant role as being widespread within the area. Wildlife surveillance is crucial to monitor for human and animal health since global distribution and flexibility in intermediate hosts of many and even more critical taeniids species may enlarge their host range.

## Highlights

• A new intermediate host for a not commonly detected tapeworm was documented.
• A new ecological niche might be hypothesized.
• Molecular investigation is crucial for the diagnosis of tapeworms to avoid misdiagnosis.
• To perform necropsy on wildlife is essential for the local and global understanding of free-living animal populations.

## Graphical abstract

**Figure:**
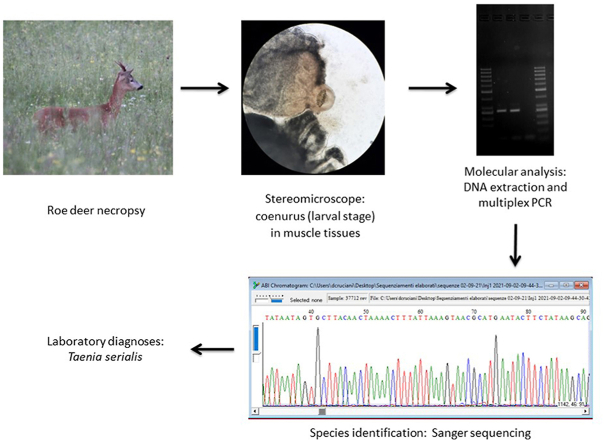

## 1 Introduction

Taeniids are multi-host parasites with an indirect life cycle that strictly depends on a predator-prey relationship. This is referred to as a multi-host trophically-transmitted parasite system (Baudrot et al., 2016). Tapeworm species, belonging to the order Cyclophyllidea, exclusively affect mammalian species in both their adult and larval stages (Hoberg, 2002), included human beings. Within this complex ecosystem predators act as definitive hosts, where parasites complete their sexual reproduction, and preys, acting as intermediate hosts, harbour the larval stage (metacestode).

Parasites with a complex life cycle may exhibit different degrees of host-specificity at each life stage. In particular taeniids, whose definitive hosts are exclusively members of the order of Carnivora, whereas intermediate hosts cover a much wider range where metacestodes can develop, such as marsupials, rodents, ruminants, and human and non-human primates (Romig et al., 2017). To measure host-specificity it is not sufficient the number of the species that a parasite can infect but also the relatedness to each other (Fredensborg, 2014; Park et al., 2018). Theoretically speaking, the phylogenetic distance between host species used by a parasite and their number can predict its ability to expand the host range by colonizing new species (Poulin et al., 2006, 2011). Trophically-transmitted parasites necessitate high predation rates assuring high transmission levels within the food chain (Parker et al., 2003), and so, a generalist parasite that uses several intermediate host species has more likelihood to infect its definitive host if this feeds broadly among many prey species (Park, 2019). In light of this, parasites using intermediate hosts and transmitted via food chain are much less specific (Poulin et al., 2006). Thus, knowing the host breadth at the different life cycle stage is a fundamental concept of the biology and epidemiology of these multi-host parasites.

Sporadic cases of metacestodoses in uncommon intermediate hosts have been reported and often they seem to be attributable to epiphenomenon due to a high parasite pressure, where the intermediate host usually behaves as dead-end host (Poglayen et al., 2016; Zaffarano et al., 2021). Other causes implied in an enlargement of the host range could be associated to environmental factors and to the abundance of host species able to be parasitized (Walker et al., 2017).

Morphological identification of tapeworms is challenging and occasionally may produce misdiagnosis. Molecular tools are crucial to understand the eco-epidemiology and to perform an accurate diagnosis in a multi-host trophically-transmitted parasite system (Schneider-Crease et al., 2013). Thus, we below describe a case of metacestodosis due to *Taenia serialis* in a roe deer that has never been previously reported as a suitable intermediate host of this parasite.

## 2 Materials and methods

### 2.1 Necropsy

A free-ranging adult male European roe deer died in mid-August 2021 in Frontignano (42°55′12″N; 13°09′26″E), municipality of Ussita, province of Macerata (Italy), within the territory of the Monti Sibillini National Park (MSNP). Before dying was spotted having movement disorders and difficulties standing up. The carcass was brought in to the Istituto Zooprofilattico Sperimentale Umbria and Marche “Togo Rosati” of Tolentino (Marche region), where a necropsy was performed to determine the causes of death.

**Fig. 1:**
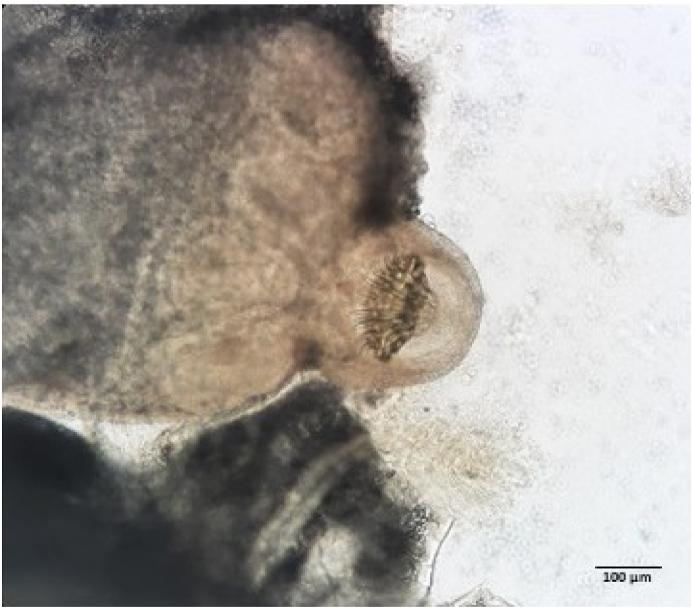
Microscope view 100 magnifications of the scolex collected from one of the cysts recovered in a wild European roe deer.

**Fig. 2:**
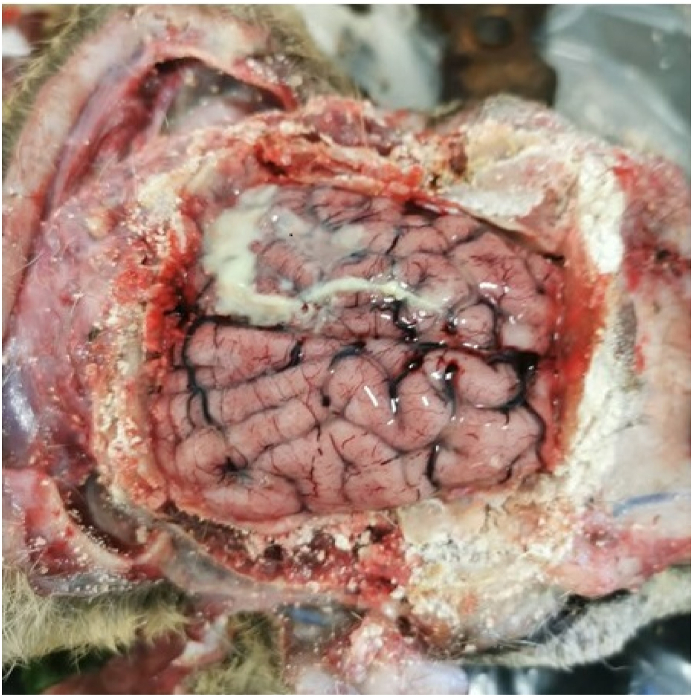
Note the meningeal vessels' congestion and the suppurative exudate covering the meningeal surface.

At necropsy, three cyst-like lesions (1 cm Ø) were accidently recovered and subsequently extracted from the surface of rectus abdominis muscle, pectoral muscle and myocardial. A cyst was carefully sectioned, posed on a slide covered with a coverslip, and then observed under a light microscope (Fig. 1). The cerebral inspection revealed congestion in the meningeal vessels, purulent exudate covering the cerebral sulcus, and the meninges appeared slightly thick (Fig. 2). Cultures for aerobic and anaerobic bacteria from swabs taken of lesion were conducted according to standard methods. The plates were incubated at 37 °C ± 1 for 48 h. The isolate was identified to species level using MALDI-TOF spectroscopy.

### 2.2 Molecular analysis

Molecular investigations were carried out for the identification of parasite. Genomic DNA was extracted from cysts using a commercial kit, according to the manufacturer's instructions (QIAamp DNA Mini Kit, fluid protocol, QIAGEN®, Valencia, CA, USA) and then amplified with a multiplex-polymerase chain reaction (m-PCR), according to Trachsel et al. (2007). Three different amplicons sizes could be obtained using distinct primer pairs: 395 bp for *E. multilocularis nad1* gene using Cest1 and Cest2, 117 bp for *E. granulosus rrnS* gene through Cest4 and Cest5 and 267 bp for *Taenia* spp. *rrnS* gene using Cest3 and Cest5. Electrophoresis on 2% agarose gel stained with Midori Green Advance (NIPPON Genetics®, Düren, Germany) was performed and PCR positive reactions were purified by QIAquick PCR Purification Kit (QIAGEN®) and then subjected to Sanger sequencing using BrilliantDyeTM Terminator v3.1 Cycle Sequencing Kit (NimaGen®), according to manufacturer's instructions and 3500 Genetic Analyzer (Applied Biosystems®, Foster City, CA, USA). Consensus sequence was created by BioEdit Sequence Alignment Editor software v 7.0.9.0 and then aligned in GenBank database. Phylogenetic analysis was performed by using the Maximum Likelihood method.

## 3 Results

**Fig. 3:**
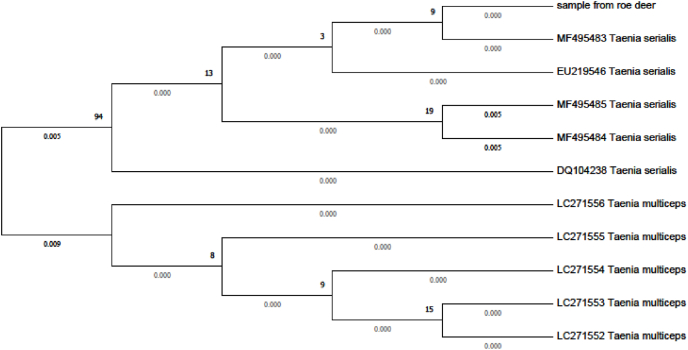
Phylogenetic relationships between two species of *Taenia* (*T. serialis* and *T. multiceps*) and the sample obtained for this study (sample from roe deer).

M-PCR gave a ~270-bp amplicon, referable to *Taenia* spp. (267 bp). Sanger sequencing showed 99% query cover, 2e-109 e-value, and 100% identity with *Taenia serialis* (GenBank accession number MF495483). Phylogenetic reconstruction confirmed sequencing results, showing that the sample obtained for this study is more closely related to *T. serialis* than to other Taeniid species, like *T. multiceps*, as shown in Fig. 3*.*

As for the intracranial abscessation-suppurative meningoencephalitis leading to death the roe deer, the bacterial culture result revealed very small white, opaque and glistening colonies with β-hemolysis, finally identified as *Trueperella pyogenes* (formerly *Arcanobacterium pyogenes*) by MALDI-TOF.

## 4 Discussion

This contribution describes the first detection, through molecular identification, of *T. serialis* in a never previously reported intermediate host, the European roe deer, and establishes a new geographic confirmation of this parasite, opening a new ecological niche potentially occupied by the tapeworm.

The larval stage produced by *T. serialis* is a coenurus closely related to *T. multiceps*. Contrary to *T. multiceps* that mainly exploits a domestic life cycle in sheep-farming regions (Morandi et al., 2020), wildlife plays a more prominent role in the *T. serialis* epidemiology (Deplazes et al., 2019). Canids act as definitive hosts, earlier reported as red foxes (Citterio et al., 2021), artic foxes (Andreassen et al., 2017) and hyena, coyote, and jackal (Deplazes et al., 2019). As regards intermediate hosts, *Taenia serialis*-coenurosis is already diagnosed in lagomorphs, rodents, and several cases in primates (Schneider-Crease et al., 2013, 2017), rarely detected also in cats, sheep (see Deplazes et al., 2019), and marsupials (Dunsmore, 1968; Hough, 2000), so far. Tissues affected with coenurus infection are classically CNS, spinal cord, and eyes when *T. muticeps* is involved, otherwise soft tissue/subcutaneous connective tissue, the musculo-skeletal system, and visceral organs if etiology is represented by *T. serialis*, *T. brauni*, and *T. glomeratus* (Lescano and Zunt, 2013).

In literature, only 11 cases of human *T. serialis*-coenurosis, based only on morphological identification, are mentioned, mostly from Africa, secondarily from North America and France (Deplazes et al., 2019). Although this low incidence may reflect a low parasite pressure or a hypothetical resistance of humans to this zoonoses, the real epidemiology of this rare parasitic disease is still unclear. Additionally, in animals few diagnoses have been confirmed by using molecular analysis for species identification that may imply *T. serialis* to be more widespread and flexible in the selection of intermediate hosts than previously hypothesized (Schneider-Crease et al., 2017). Furthermore, Deplazes et al. (2019), in a review, report cerebral infection of *T. serialis* in sheep without giving details on how the diagnoses were performed, after that, any other author does not describe *T. serialis* in ungulate species, neither domestic or free-living.

To our knowledge, few published articles report the presence of *T. serialis* either in definitive or intermediate hosts in Italy. Recently, Citterio et al. (2021) detected *T. serialis* eggs in only 0.2% of 2872 red foxes collected from 2012 to 2018 in Northern Italy. Interestingly, Italian wolf (*Canis lupus italicus*), although is a deeply studied species, where hundreds of researches investigate its helminths, has never been diagnosed positive for *T. serialis*, also considering different regions, ecological setting (anthropic *vs* not anthropic), and sample size (see for example: Gori et al., 2015; Poglayen et al., 2017; Macchioni et al., 2021), making the red fox the first putative definitive host. In support of this, preliminary results, obtained by examining guts of some wolves found dead in the same area, have not still revealed the presence of *T. serialis* tapeworm (unpublished data). However, its life cycle seems to be completely related to a wild/free-ranging hosts system predominantly consisting of wild canids, and lagomorphs and rodents (Deplazes et al., 2019), and roe deer has accidently fallen into it. Anyway, the roe deer cannot be merely considered as an “accidental” intermediate host, since the metacestode, being fertile (with scolex) as shown in Fig. 1, would potentially infect a definitive host. As the three cyst-like lesions were small and similar in size, we can hold that there was a single time of exposure, and they did not clinically affect the host making our finding an incidental report.

Adding an intermediate-prey host into the cycle of trophically-transmitted parasites is profitable when the intermediate host density is higher than the definitive host density, and a high predation rate with a low mortality rate in the intermediate host species are present (Choisy et al., 2003). Considering these assumptions, MSNP is an excellent environment where establishing an endemic cycle as another deer species potentially susceptible, the red deer, is present within the area. Although *T. serialis* is not a conservation issue, economic concerns may emerge for hunters and meat producers, related to the damage of carcasses by coenuri, as well as it may happen for other matacestodoses affecting specifically cervids, for example *T. krabbei* (Formenti et al., 2017), even if is never reported in the area.

Contextually, as regards the real causes of death, the roe deer has showed evidence of cerebral disorders (*in vitam*), later confirmed by necropsy, where an intracranial abscessation-suppurative meningoencephalitis was noticed. The bacteria responsible for the suppurative lesion has been identified as *T. pyogenes* based on MALDI-TOF identification. This gram-positive opportunistic bacterial pathogen resides on the skin layer of deer; according to Karns et al. (2009), in North America (Maryland), can account for 35% of annual mortality of mature males, as a consequence of infected wounds acquired through frontal head injuries during intra-species fights occurring in the breeding season as it was for the reported case.

## 5 Conclusion

A necropsy supported by molecular analysis confirmed the European roe deer from the MSNP as a suitable intermediate host for *T. serialis*. Despite its life cycle belongs predominantly to sylvatic environment, the epidemiology of *T. serialis* is not yet definitely understood. Although an endemic scenario is still not defined within the MSNP, we may hypothesize that the roe deer acquired infection most likely from red foxes as suitable definitive hosts widely present in the area, and secondarily from Italian wolves. Anyway, further studies exploring for the main definitive host(s) in the same study area are urgently needed. By combining necropsy and laboratory analysis is easier to reach a complete and accurate diagnosis and shed light on uncommon parasitic diseases. The introduction of roe deer as intermediate host in an area where the taxonomically related red deer is present may open an important chapter about the *T. serialis* ecology. In conclusion, the ecology of multi-host trophically-transmitted parasites is continuously dynamic since depends on dynamic systems as predator-prey interactions are, thus new potential niches for these parasites cannot be totally excluded. Wildlife surveillance is crucial to monitor for human and animal health since global distribution and flexibility in intermediate hosts of many and even more critical taeniids species may enlarge their host range.

## Declaration of competing interest

All authors declare that they have no competing interests.
